# Asthma management in low and middle income countries: case for change

**DOI:** 10.1183/13993003.03179-2021

**Published:** 2022-09-15

**Authors:** Kevin Mortimer, Helen K. Reddel, Paulo M. Pitrez, Eric D. Bateman

**Affiliations:** 1 Dept of Respiratory Medicine, Liverpool University Hospitals NHS Foundation Trust, Liverpool, UK; 2 Dept of Medicine, University of Cambridge, Cambridge, UK; 3 The Woolcock Institute of Medical Research and The University of Sydney, Sydney, Australia; 4 Pediatric Respiratory Division, Hospital Moinhos de Vento, Porto Alegre, Brazil; 5 Division of Pulmonology, Dept of Medicine, University of Cape Town, Cape Town, South Africa

## Abstract

Asthma is the most common noncommunicable disease in children, and among the most common in adults. The great majority of people with asthma live in low and middle income countries (LMICs), which have disproportionately high asthma-related morbidity and mortality. Essential inhaled medications, particularly those containing inhaled corticosteroids (ICS), are often unavailable or unaffordable, and this explains much of the global burden of preventable asthma morbidity and mortality. Guidelines developed for LMICs are generally based on the outdated assumption that patients with asthma symptoms <1–3 times per week do not need (or benefit from) ICS. Even when ICS are prescribed, many patients manage their asthma with oral or inhaled short-acting β_2_-agonists (SABA) alone, owing to issues of availability and affordability. A single ICS–formoterol inhaler-based approach to asthma management for all severities of asthma, from mild to severe, starting at diagnosis, might overcome SABA overuse/over-reliance and reduce the burden of symptoms and severe exacerbations. However, ICS–formoterol inhalers are currently very poorly available or unaffordable in LMICs. There is a pressing need for pragmatic clinical trial evidence of the feasibility and cost-effectiveness of this and other strategies to improve asthma care in these countries. The global health inequality in asthma care that deprives so many children, adolescents and adults of healthy lives and puts them at increased risk of death, despite the availability of highly effective therapeutic approaches, is unacceptable. A World Health Assembly Resolution on universal access to affordable and effective asthma care is needed to focus attention and investment on addressing this need.

## Abstract 2

**Many children, adolescents and adults with asthma in LMICs lack access to effective and affordable asthma care that would improve and save lives. A World Health Assembly Resolution on access to effective and affordable asthma care for all is needed.** https://bit.ly/3gLquDa

## Introduction

**FIGURE 1:**
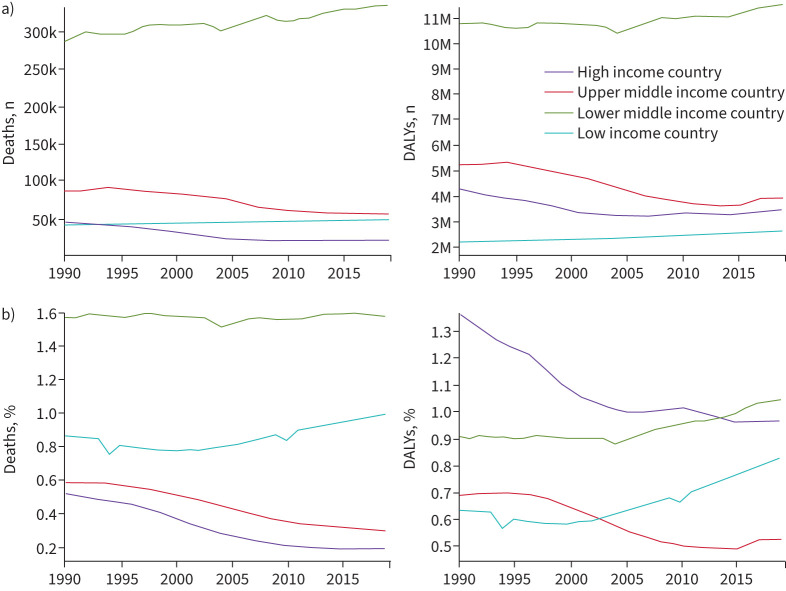
Deaths and disability due to asthma 1990–2019. a) Number of deaths and disability-adjusted life years (DALYs) due to asthma according to World Bank income category for both sexes and all ages. Between 1990 and 2019 the number of deaths and DALYs in lower middle income countries far exceeded those of other income brackets. b) Percentage of deaths and DALYs due to asthma according to World Bank income category for both sexes and all ages. In 2019, rates of asthma deaths were highest in lower middle income countries, followed by low income countries. By 2019, disease burden (DALYs) in lower middle income countries had overtaken that of high income countries. Both death rates and DALYs have been increasing in low income countries, and DALYs in lower middle income countries. In contrast, deaths and disease burden have decreased steadily in high income countries from 1990 to 2019. Adapted from [6].

Asthma is the most common noncommunicable disease in children, and among the most common in adults [1]. According to the most recent estimates from the Global Asthma Network Phase I study, around one in 10 children and adults have symptoms of asthma and one in 20 school-aged children have severe asthma symptoms, with marked variations in prevalence and in prevalence trends between countries and regions of the world [2–4]. The Global Burden of Disease Study estimated that asthma caused the loss of 21.6 million healthy years of life (disability-adjusted life years) and 461 069 deaths in 2019 [5, 6]. Approximately 90% of the asthma burden of disease is borne by people living low and middle income countries (LMICs) [7]. Some countries report very high (up to 90%) rates of uncontrolled asthma [8–10]. While the prevalence of asthma is highest in countries with a high Socio-Demographic Index (SDI), death rates from asthma are highest in countries with low and lower middle incomes (figure 1) [6].

### What is different about asthma in LMICs?

The Global Initiative for Asthma (GINA) describes asthma as a heterogeneous disease usually characterised by chronic airway inflammation, and defines it by a “history of respiratory symptoms such as wheeze, shortness of breath, chest tightness and cough that vary over time and in intensity, together with variable expiratory airflow limitation” [11]. Countries currently classified by the World Bank as low income (27 countries) or lower middle income (55 countries) economies include most countries in sub-Saharan Africa and in South Asia and several in the Middle East and North Africa, East Asia and Pacific, Latin America and Caribbean, and Europe and Central Asia regions [12]. A further 55 countries are classified as upper middle income economies [13].

There is no logical biological rationale for asthma to differ between countries according to their income alone. The substantial observed differences in asthma burden of disease with respect to national income (figure 1) [7, 14] probably reflect a range of factors, including differences in asthma prevalence and asthma management. Differences in asthma prevalence may be due to early-life exposures, clinical presentation, differential diagnosis, clinical course of asthma and comorbidity rates.

Environmental exposures *in utero* or in early life that are associated with increased risk of developing asthma include tobacco smoke [15, 16], vitamin D insufficiency [17], paracetamol [16, 18, 19], broad-spectrum antibiotics [16, 20, 21], allergens [22–24], obesity [16, 25, 26], air pollution [16, 27–30] and childhood respiratory infections [16, 31]. Dietary factors [32–34], certain bacterial exposures [35–37], helminth infection [38] and microbiome diversity [39, 40] may be protective. While tobacco use has decreased in LMICs since 2000 [41], in these countries higher smoking rates have been reported among men and women in lower socioeconomic groups [42], suggesting that *in utero* and infant exposures are likely highest among the poorest of the poor. Increasing urbanisation in LMICs may lead to changes in diet, parasitic infections and exposure to air pollution, which might help explain changing trends in the prevalence of asthma [43, 44].

Differences in burden of disease are likely also to be attributable to suboptimal asthma treatment due to socioeconomic deprivation. The cost of medicines is a major challenge for LMICs [45]. Not only are newer asthma treatments and approaches often unavailable, assumed to be unaffordable, or available only at some levels of the health system, but there is also a lack of research on their effectiveness and implementation feasibility in these populations. The widening care gap between rich and poor, north and south, highlights an urgent need to reassess assumptions about “best buys” for asthma care in resource-constrained countries and regions.

Other reasons for suboptimal asthma outcomes differ between countries, so generalisations based on national income should be avoided. It is more helpful to identify the key barriers to effective asthma care in each country, and address them systematically. Barriers may include funding models based on a mix of private and public sources favouring a small, affluent segment of the population, organisation of health services designed for acute care at the expense of chronic and preventive care, or community disinclination to accept optimal asthma treatment. In some countries, cultural factors (*e.g.* health beliefs that result in unwillingness to accept the diagnosis of asthma, religious practices and beliefs, roles of traditional health workers, reliance on home remedies or unsupported beliefs about asthma medication risks) may present major obstacles to optimal asthma care [46–50]. Without an understanding of these key barriers, the solutions that are offered by well-meaning agencies may not achieve their objectives.

### Scope of this review

In this review, we examine current recommendations for asthma care from several organisations working in LMICs, strategies for improving care access and quality in these regions, and the challenges and opportunities for clinicians and healthcare systems translating recent advances in asthma care into actionable initiatives. We identify research questions and propose health service priorities to address the global inequalities in access to basic, effective asthma care for children, adolescents and adults with asthma living in LMICs.

## Primary prevention of asthma

While effective approaches to primary prevention of asthma have not yet been established [11], GINA recommends environmental tobacco smoke avoidance for pregnant women and infants, sufficient vitamin D levels before and during pregnancy for women with asthma, vaginal delivery where possible and avoidance of broad-spectrum antibiotics during the first year of life [11].

Higher cigarette pricing may be an effective deterrent to youth smoking [51], and nicotine replacement therapy and counselling appear effective in smoking cessation in LMICs [52], but there is limited rigorous research on other smoking cessation interventions in these regions [52].

If enough evidence emerges to support the use of preventive interventions within national health programmes (*e.g.* routine prenatal vitamin D supplementation for pregnant women), it is crucial to ensure these can be implemented in LMICs.

## Diagnosis of asthma

Asthma is a clinical diagnosis, based on a history of characteristic symptom patterns and evidence of variable expiratory airflow limitation [11]. In well-resourced healthcare settings, this evidence can be derived from spirometry with bronchodilator responsiveness (“reversibility”) testing or bronchial provocation challenge testing. In LMICs, even when available, these investigations are still substantially underused (either because they are unaffordable [53] or because they are time-consuming in busy clinics), and more than one test is often required to confirm airflow variability [11].

In LMICs, where there is typically limited availability of these diagnostic technologies, and where the differential diagnosis of asthma may include endemic respiratory disease (*e.g.* tuberculosis, HIV/AIDS and parasitic or fungal lung diseases), clinicians place greater reliance on clinical findings and often use syndromic approaches to diagnosis and management [1, 11].

The principle of syndromic diagnosis is pattern recognition at the cost of precision, on the assumption that the condition is under-diagnosed and under-treated, which is valid for most LMICs. However, syndromic diagnosis can lead to under-treatment or inappropriate treatment of asthma [54], and may result in overdiagnosis and overtreatment in developed countries [55, 56].

Structured approaches to primary care diagnostic assessment of patients presenting with respiratory symptoms form part of several strategies that have been developed for improving respiratory disease management in LMICs (table 1) [57–66], particularly in countries with high prevalence of tuberculosis.

**TABLE 1:** Current strategies for improving diagnosis and management of respiratory disease in primary care in low and middle income countries (LMICs)

**Strategy**	**Objectives**	**Relevance to asthma**
**Practical Approach to Lung Disease (PAL) developed by WHO Stop TB Partnership (www.stoptb.org)**	Improve respiratory care in primary health and improve coordination and integration of respiratory case management in LMICs (*e.g.* with TB and HIV programmes) [57]	Outcomes reported in LMICs include increase in asthma diagnosis, reductions in hospitalisations/emergency room visits and symptoms, and improved asthma treatment (*e.g.* increased use of ICS, reduction in oral SABA, theophylline) [57, 58]
**WHO Package of essential noncommunicable (PEN) disease interventions for primary care [58–6** **0** **]**	Provide technical guidance (*e.g.* protocols and tools) for managing noncommunicable diseases in primary care and prioritise cost-effective interventions that can be delivered to an acceptable quality of care, even in resource-poor settings [59]	Includes algorithm for assessment, diagnosis and management of chronic respiratory diseases applicable to patients presenting with cough, breathing difficulty, tight chest and/or wheezing, and recommendations for essential drugs and technologies [59]
**WHO Integrated Management of Childhood Illness (IMCI) strategy [** **61, 62** **]**	Reduce child mortality and morbidity in developing countries by combining improved management of common childhood illnesses with proper nutrition and immunisation [63]	Includes algorithm for assessment when child presents with cough or difficulty breathing (asthma is a consideration when pneumonia and respiratory tract infections ruled out in child with chronic cough) [62]
**WHO Integrated Management of Adolescent and Adult Illness (IMAI) [64]**	Provide effective clinical approach and protocols for the management of common and serious or potentially life-threatening conditions in adolescents and adults within district hospitals in resource-constrained settings, including limited essential drugs, laboratory tests and equipment	Includes guides to assessment and management of cough and shortness of breath, and an approach to the severely ill patient with difficulty breathing (including recognition and management of acute bronchospasm)
**Practical Approach to Care Kit (PACK) developed by Knowledge Translation Unit, University of Cape Town Lung Institute [65]**	Simplify and standardise care delivered by primary health workers in South Africa, Botswana, Nigeria, Ethiopia and Brazil	Clinical decision support tool (the PACK guide) includes section on chronic respiratory disease [66]

WHO: World Health Organization; TB: tuberculosis; ICS: inhaled corticosteroids; SABA: short-acting β_2_-agonists.

Both GINA [11] and the World Health Organization (WHO) package of essential noncommunicable (PEN) disease interventions for primary care [59, 60] advise that the presence of variable expiratory airflow limitation (including reversible obstruction) can be confirmed by peak expiratory flow (PEF) where spirometry is not available. WHO PEN lists the PEF meter as an essential tool in the management of chronic respiratory diseases and proposes its use in support of a clinical diagnosis based on a patient's medical history, where a ≥20% improvement in PEF 15 min after giving two puffs of salbutamol increases the likelihood of a diagnosis of asthma *versus* chronic obstructive pulmonary disease (COPD) and other diagnoses [59, 60]. As an alternative, GINA advises that documenting symptoms and PEF before and after a therapeutic trial with as-needed short-acting β_2_-agonist (SABA) and regular inhaled corticosteroids (ICS), with a 1-week course of oral corticosteroids (OCS) if necessary, can help to confirm the diagnosis of asthma (or prompt investigation for alternative diagnoses) before starting long-term controller treatment [11].

## Management of asthma

The fundamental principles and aims of asthma treatment [11] are the same in LMICs as in high income countries (HICs), but common barriers to effective long-term asthma care include the lack of availability and affordability of inhaled medicines, and prioritisation of acute care over chronic care by healthcare systems.

### Medication recommendations for long-term management

Asthma guidelines almost universally recommend a stepwise approach to medication to achieve and maintain good asthma symptom control and prevent asthma exacerbations. However, some older treatment guidelines used in LMICs do not take this approach [67].

Central to asthma management is the use of ICS to improve symptom control and reduce the risks of exacerbations and asthma deaths [11], together with a bronchodilator inhaler for rapid symptom relief. ICS reduce the risk of asthma mortality by over 50% [68], reduce the risk of hospitalisation by 31% [69] and reduce the risk of serious exacerbations by almost half [70], with most of the benefit achieved with a low dose [71].

However, in recommendations developed for chronic asthma management in LMICs [59, 72, 73], treatment starts with as-needed salbutamol (SABA), adding low-dose ICS if initial symptoms are more than once [72] or twice [59] a week. Then, if asthma is still uncontrolled, the ICS dose is increased [59, 72], adding oral theophylline and/or prednisone [59] or prescribing salbutamol to be taken at regular intervals rather than as needed [72].

These recommendations differ substantially from longstanding global evidence-based recommendations, owing to guideline developers’ awareness of the limited availability and affordability of medicines in LMICs. In particular, the addition of a long-acting β_2_-agonist (LABA), preferably in a combination ICS–LABA inhaler, has long been preferred over high-dose ICS monotherapy because of the greater efficacy and better safety profile.

Some recommendations in LMIC guidelines (table 2) [59, 72, 74] are based on very slender evidence, such as oral theophylline [59] at Step 4, which is no longer recommended by GINA (table 3) owing to weak evidence of benefit and high risk of adverse effects [11]. Further, the cost of different asthma medicines varies widely between LMICs, so a single recommendation at each step may not be the best option given the need to take cost and affordability into account.

**TABLE 2:** Comparison of asthma treatment steps in guidelines developed for low and middle income countries

**Level of treatment^#^**	**WHO PEN** [59]	**MSF** [72]	**Union** [74]
**Mild asthma**	*Step 1* As-needed inhaled salbutamol	*Intermittent asthma* As-needed inhaled salbutamol	*Intermittent asthma* As-needed inhaled salbutamol
	*Step 2* Regular low-dose inhaled beclometasone (starting dose: adults 100 μg twice daily; children 100 μg once or twice daily) PLUS as-needed inhaled salbutamol	*Mild persistent asthma* Regular inhaled beclometasone^¶^ PLUS as-needed inhaled salbutamol	*Mild* HFA beclometasone 100 μg, 2 puffs per day PLUS as-needed inhaled salbutamol
	*Step 3* Regular medium-dose inhaled beclometasone (adults: 220 μg or 400 μg twice daily) PLUS as-needed inhaled salbutamol	*Moderate persistent asthma (symptoms daily)* Regular inhaled beclometasone^¶^ PLUS inhaled salbutamol 4 times daily	*Moderate* HFA beclometasone 100 μg, 4 puffs per day PLUS as-needed inhaled salbutamol
**Severe asthma**	*Step 4* Add low-dose oral theophylline *Step 5* Add low-dose oral prednisolone (*e.g.* <10 mg)	*Severe persistent asthma* Inhaled beclometasone^¶^ PLUS inhaled salbutamol 4–6 times daily	*Severe* HFA beclometasone 100 μg, 8 puffs per day PLUS as-needed inhaled salbutamol

WHO PEN: World Health Organization package of essential noncommunicable disease interventions for primary care; MSF: Médecins Sans Frontières; Union: International Union Against Tuberculosis and Lung Disease; HFA: hydrofluoroalkane-134a. ^#^: increasing intensity according to severity (mild to severe). ^¶^: doses depend on asthma severity: children 50–100 μg twice daily and increase to 200 μg twice daily if necessary (up to maximum 800 μg daily); adults 100–250 μg twice daily and increase to 500 μg twice daily if necessary (up to maximum 1500 μg daily).

**TABLE 3:** Summary of Global Initiative for Asthma treatment steps (2021)

**Step**	**Children ≤5** **years**	**Children 6–11** **years**	**Adolescents and adults**	
			**Track 1 (preferred)**	**Track 2 (non-preferred alternative)** **^#^**
**1**	As-needed SABA alone if intermittent viral wheezing, with no/few interval symptoms	Low-dose ICS taken whenever as-needed SABA is taken	As-needed low-dose ICS–formoterol	Low-dose ICS taken whenever as-needed SABA is taken
**2**	Daily low-dose ICS^¶^ PLUS as-needed SABA	Low-dose maintenance ICS^¶^ PLUS as-needed SABA	As-needed low-dose ICS–formoterol	Low-dose maintenance ICS PLUS as-needed SABA
**3**	Double “low-dose” ICS^¶^ PLUS as-needed SABA	Low-dose maintenance ICS–LABA^¶^ OR medium-dose maintenance ICS OR very low-dose ICS–formoterol as MART	MART: low-dose maintenance ICS–formoterol PLUS as-needed low-dose ICS–formoterol	Low-dose maintenance ICS–LABA PLUS as-needed SABA
**4**	Continue controller^¶^ and refer for specialist assessment	Medium-dose ICS–LABA^¶^^,+^ OR low-dose ICS–formoterol as MART Refer for expert advice	MART: medium-dose maintenance ICS–formoterol PLUS as-needed low-dose ICS–formoterol	Medium/high-dose maintenance ICS–LABA PLUS as-needed SABA
**5**	(No step 5)	As for Step 4 PLUS referral ±higher-dose ICS–LABA ±add-on biologics^§^	As for Step 4 PLUS add-on LAMA Referral for phenotypic assessment±biologics^§^ Consider high-dose ICS–formoterol	As for Step 4 PLUS add-on LAMA Referral for phenotypic assessment±biologics^§^ Consider high-dose ICS–LABA

Information from Boxes 3–5A, 3–5B, 6–5 [11]. SABA: short-acting β_2_-agonist (salbutamol or terbutaline); ICS: inhaled corticosteroids; MART: maintenance and reliever therapy; LABA: long-acting β_2_-agonist; LAMA: long-acting muscarinic antagonists. ^#^: before considering this track, check if the patient is likely to be adherent with daily controller. ^¶^: for children 6–11 years, leukotriene receptor antagonists can be considered as an alternative (Step 2) or additional (Steps 3+) controller (see Box 3–6 and Box 6–6 [11] for details and ICS doses). ^+^: tiotropium can be added. ^§^: consider add-on biologic (*e.g.* anti-immunoglobulin E therapy, anti-interleukin (IL)5 or anti-IL5 receptor therapy or anti-IL4 receptor therapy).

**FIGURE 2:**
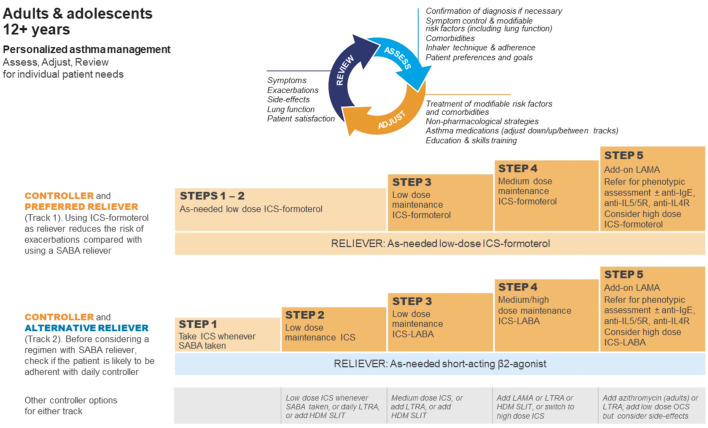
Global Initiative for Asthma (GINA) recommendations for management of asthma in adolescents and adolescents. GINA treatment steps for adults and adolescents are divided into two tracks, depending on the inhaled reliever medication. Within Track 1 (preferred approach), low-dose inhaled corticosteroid (ICS)–formoterol is the reliever at all steps. Within track 2 (alternative approach), short-acting β_2_-agonist (SABA) is the reliever at all steps. LAMA: long-acting muscarinic antagonist; Ig: immunoglobulin; IL: interleukin; LABA: long-acting β_2_-agonist; LTRA: leukotriene receptor antagonist; HDM: house dust mite; SLIT: sublingual immunotherapy; OCS: oral corticosteroids. Reproduced from [11] with permission.

Differences between LMIC-specific recommendations and GINA recommendations increased further in 2019, when GINA recommended against treating asthma in adults and adolescents with SABA alone, and recommended that conventional ICS regimens with a SABA reliever should only be used if the patient is likely to be adherent (table 3 and figure 2) [75]. These changes were based on evidence of the risks associated with SABA-only treatment and SABA overuse, the almost universally poor adherence with maintenance ICS in the community, and the findings of large new studies of as-needed combination ICS–formoterol in patients with mild asthma. These showed that the risk of severe exacerbations was reduced by ∼60% compared with SABA alone, with similar benefit for symptoms, lung function, airway inflammation and exacerbations as regular ICS, without the requirement for daily treatment [11, 76].

### Managing acute asthma exacerbations

Asthma is often still regarded as an acute disease by patients in LMICs [77]. Health services may reinforce this perception by providing widely available and relatively affordable emergency room treatment but with little or no follow-up. Patients and physicians may consider repeated attendances at emergency facilities as the normal and inevitable consequence of being “asthmatic”.

GINA recommendations [11] for the assessment of acute asthma exacerbations include pulse oximetry, and PEF for patients aged ≥5 years as an alternative to spirometry. These are readily available in LMICs and, as mentioned above, PEF meters are listed among WHO PEN essential technologies and tools (with disposable mouthpieces also required), with pulse oximeters recommended “when resources permit” [59].

While escalation to critical care facilities for acute severe asthma not responding to medical treatment is often not an option in LMICs, medicines (oxygen, inhaled/nebulised bronchodilators, OCS and parenteral adrenaline/epinephrine and magnesium sulfate) are usually available. However, oxygen supplies may be an issue owing to demand during the coronavirus disease 2019 (COVID-19) pandemic [78].

### Medication prioritisation

Recommendations by WHO and the International Union Against Tuberculosis and Lung Disease (the Union) [79] are widely consulted and form the basis of treatments offered in many, if not most, LMICs. The WHO Model List of Essential Medicines (table 4) [80, 81] is considered as the “best buys” for asthma care. The Union's 2020–2025 strategic plan emphasises the need for affordable, regular access to inhaled bronchodilators and ICS using appropriate devices for people with asthma [79].

**TABLE 4:** Medications for asthma in the World Health Organization Model List of Essential Medicines

**First listed medicine in category**	**Dose^#^**
**Budesonide**	Inhalation: 100 μg per dose; 200 μg per dose
**Budesonide+formoterol**	Dry-powder inhaler: 100 μg+6 μg per dose; 200 μg+6 μg per dose
**Epinephrine (adrenaline) injection**	1 mg·mL^−1^ (as hydrochloride or hydrogen tartrate) in 1 mL ampoule
**Ipratropium bromide**	Inhalation: 20 μg per metered dose
**Salbutamol**	Inhalation: 100 μg (as sulfate) per dose
	Injection: 50 μg·mL^−1^ (as sulfate) in 5 mL ampoule
	Metered dose inhaler: 100 μg (as sulfate) per dose
	Respirator solution for use in nebulisers: 5 mg·mL^−1^ (as sulfate)
**Tiotropium**	Powder for inhalation, capsule: 18 μg
	Inhalation solution: 1.25 μg; 2.5 μg per actuation

Information from [80, 81]. ^#^: metered doses shown, see product information for delivered doses.

Inhaled asthma medicines can be delivered *via* a pressurised metered-dose inhaler (pMDI) or dry-powder inhaler. GINA, WHO, Médicins Sans Frontières and Union guidelines each emphasise the importance of checking inhaler technique regularly.

WHO recommends that all pMDIs should be issued with spacer devices [59] in recognition of the high prevalence of poor inhaler technique, which results in suboptimal dose delivery and asthma outcomes. This might be even more important in children. Spacers are included in the WHO list of essential technology, but the recommendation is seldom followed because of obstacles to the purchase or manufacture of spacers, practical issues of cleaning and inconvenience for ambulatory use. Effective spacers can be made at no cost from plastic drink bottles [82].

Medicines selected as “essential” are not necessarily the most effective or convenient, particularly for patients with more severe disease, and a limited choice does not allow for consideration of patient preferences and likelihood of adherence. However, ICS-containing controllers, when provided for large populations, have achieved impressive reductions in mortality and morbidity [83], reflected in decreasing asthma deaths in HICs seen from 1990 (figure 1). In Brazil, government policy ensuring nationwide easy access to ICS, at no cost to patients, was associated with a 34% reduction in hospitalisations for asthma [84].

### Limited access to essential quality-assured asthma medicines in LMICs

Inclusion of essential asthma medicines in formularies and guidelines does not assure sustained and equitable supply to patients. The supply of medicines to the point of delivery to patients in many LMICs tends to be sporadic for a wide variety of reasons, sometimes determined by the ability of governments to pay for supplies, issues relating to procurement, poor administration and record keeping and problems in the supply chain, particularly to remote dispensaries.

Availability of asthma medicines varies widely between LMICs, ranging from those with accessible health services offering long-term low- or medium-dose ICS plus a SABA as reliever, to those that provide oral bronchodilators (salbutamol and theophylline tablets/solutions) supplemented from time to time with OCS. Oral bronchodilators have a slow onset of action and a higher rate of adverse effects than inhaled SABA, and even occasional courses of OCS are associated with a significant risk of short-term adverse effects such as pneumonia and sepsis [85], and with long-term adverse effects including osteoporosis, cataract and diabetes [86].

Patterns of access to asthma medicines can be recognised across LMICs and fall into four categories: 1) countries with no regular secure access to inhaled asthma treatments, characterised by fragmentary health services, reliance on traditional medicine or private practitioners, and pervasive poverty with reliance on grants and philanthropy; 2) countries with rudimentary primary care services but where ICS can only be obtained through private purchase, making them inaccessible to most of the population due to prohibitive cost; 3) countries with functioning government-funded primary care services, but limited access to medicines because asthma medications are a lower priority owing to competing burdens of other diseases; and 4) countries where asthma medicines are available through functioning government-funded primary care services, but there are significant barriers to access, long queues (high demand), transport costs and competing life priorities.

The largest survey (52 countries) of the accessibility and affordability of inhaled asthma medicines, conducted in 2011, reported that ICS were available in fewer than one in five public pharmacies and salbutamol was available in half of public hospitals [87]. ICS inhalers were unavailable in Burundi, Cameroon, Congo, Djibouti, Ecuador, Haiti, Mauritania, Myanmar, Nigeria, Pakistan, Syria, Tanzania, Togo and Vietnam, despite their listing by WHO as essential medicines [87].

Obtaining asthma medicines often represents a catastrophic household expense. In 12 countries in the 2011 survey, ICS available for purchase in pharmacies cost more than 2 days’ wages for those on the lowest salaries and in four countries more than 1 week's wages [87]. More recent surveys in individual countries provide little evidence that this situation has improved [88–90].

Although generic ICS and ICS–LABA inhalers are sometimes available, there are many more challenges for achieving bioequivalence with an inhaler than with tablets or capsules, because of the impact of factors such as the type of plastic and shape of the pMDI nozzle on medication delivery. Clinicians in LMICs often express concerns about a perceived lack of efficacy of generic inhalers. The stringency of regulatory requirements for bioequivalence of inhaled medications may vary between countries [91]. Initiatives to improve access to asthma inhalers must also ensure quality-assured products.

The COVID-19 pandemic has highlighted wider issues in relation to equitable access to affordable quality-assured health interventions in LMICs, including access to vaccinations to prevent COVID-19 [92, 93]. It is important that people with asthma in LMICs have access to these and other vaccinations relevant to protecting lung health and preventing asthma exacerbations.

## Strategies to improve asthma care in LMICs

### Integrated care strategies

Health systems in LMICs are in general more oriented towards acute care than chronic care, with a focus on maternity care and communicable diseases that have dominated historically, and on prevention programmes for endemic infections with vertical transmission (*e.g.* tuberculosis and HIV). An important consequence of the disparity between provision of long-term and acute care for asthma is high levels of avoidable morbidity and mortality due to failure to control the underlying inflammatory disease process.

The integrated strategies for primary healthcare in LMICs shown in table 1 [57, 59–65] were developed to improve chronic disease management as well as acute care. Their success depends on embedding the guidance as usual practice within organised healthcare systems. This requires the acceptance and commitment of health authorities sustained supplies of diagnostic equipment and recommended treatments ongoing training and empowerment of primary healthcare workers to ensure their effectiveness and sustainability and acceptance and adherence by patients. The strategies need to be customised to the specific practice contexts of the country or region aligned with local policies and resources and continually updated. There is evidence that when properly implemented these approaches are effective in improving asthma diagnosis and treatment [84, 94, 95]. However, they can be compromised by changes in government policy and health funding priorities by lack of access to affordable medicines and by poor adherence to medication [96, 97].

The Practical Approach to Care Kit (PACK) [65, 98] which was developed in South Africa and has been implemented in several LMICs [66, 99–101], has advantages over strategies with more limited scope. PACK provides an integrated clinician support tool or guide that covers most of the conditions for which patients consult primary care clinicians, is customised to each country setting and fully integrates the management algorithms within one document, with care to ensure that all recommendations are evidence-led and conform to the needs and directives of local health authorities. It is updated almost annually to accommodate the frequent changes in recommendations in each country, with separate versions for children aged 0–12 years [102], adolescents and adults [65, 98]. This approach ensures that, while asthma is only one among the many conditions included, it gains equal emphasis in management algorithms as more pressing and common conditions that compete for the attention of policymakers and clinicians.

Several studies have evaluated integrated asthma strategies in LMICs [57]. A comprehensive approach to severe asthma management and free, easy access to essential medicines for asthma provided from the Federal Government in Brazil decreased the rate of asthma hospital admissions and markedly reduced costs to the public healthcare system and to patients [84, 103]. In Thailand, a programme of clinics run by primary care physicians across 900 hospitals, facilitating teamwork between doctors, nurses and pharmacists, markedly increased the rate of ICS use and improved asthma control [104]. HICs also provide successful models for improving asthma that may be translatable to LMICs. Data from Finland demonstrate that asthma costs can be reduced by improving and integrating care across all levels of the health system, and with early anti-inflammatory treatment [105, 106]. Syndromic diagnosis may increase the detection of respiratory diseases and diagnosis of asthma and ensure appropriate treatment [54, 107].

### Alternative evidence-based approach to asthma medication

Given the challenges of asthma management in LMICs, it is worth considering the potential benefits of introducing the single-inhaler-based approach to asthma management for all severities of asthma from mild to severe.

Although ICS use is promoted in all guidelines developed specifically for LMICs, they generally start with SABA-only treatment instead of ICS, based on the historical assumption that patients with asthma symptoms fewer than 1–3 times/week do not need (or benefit from) ICS. This, together with the dual pressures of cost and patient preference, often result in treatment continuing with SABA alone, even when ICS is prescribed. This over-reliance on SABAs and underuse of ICS is endemic in most LMICs despite the best efforts of those involved in asthma care in these settings. Further, regular use of SABAs (four times daily) is recommended in some guidelines [72], despite increasing evidence for markedly increased morbidity and mortality associated with frequent SABA use across treatment steps [108].

There is now strong evidence, across the range of asthma severity, that the use of a combination inhaler containing an ICS and formoterol (a rapid-onset LABA) as needed for symptom relief, with or without maintenance ICS–formoterol, reduces the risk of severe asthma exacerbations compared with use of a SABA reliever, with similar levels of day-to-day symptom control, and at a lower overall dose of ICS. In moderate-to-severe asthma, a recent systematic review and meta-analysis evaluating ICS–formoterol maintenance and reliever therapy (MART) identified 16 randomised clinical trials involving 22 748 participants. It found that MART reduced the risk of severe asthma exacerbations compared with the same maintenance dose of ICS–LABA (relative risk (RR) 0.68, 95% CI 0.58–0.80) and with a higher dose of ICS–LABA (RR 0.77, 95% CI 0.60–0.98) [109]. In pragmatic open-label studies, MART also reduced the risk of severe exacerbations compared with conventional best practice, including a SABA reliever [110, 111].

In mild asthma, a recent Cochrane review that included five trials of as-needed ICS–formoterol found a large reduction in severe exacerbations compared with as-needed SABA alone (OR 0.45, 95% CI 0.34–0.60) and a reduction in emergency department visits and hospitalisations compared with maintenance ICS with as-needed SABA (OR 0.63, 95% CI 0.44–0.91) [112]. The benefit with as-needed ICS–formoterol compared with maintenance ICS was independent of baseline patient characteristics, including type 2 high or low phenotypes [113, 114]. A further study in mild asthma demonstrated a similar reduction in airway hyperresponsiveness (based on exercise challenge) with as-needed ICS–formoterol as with regular low-dose ICS [115]. Consequently, ICS–formoterol therapy is now considered by GINA to be the preferred option for all severities of asthma [11, 76]. All the evidence in mild asthma to date is with budesonide–formoterol; MART is approved in many countries with both budesonide–formoterol and beclometasone–formoterol.

With budesonide–formoterol already listed on the WHO Model List of Essential Medicines [80], and manufactured by several pharmaceutical companies, these developments provide the opportunity for ICS–formoterol to become the first and central treatment for patients diagnosed with asthma. From a patient perspective, there are several potential advantages of this approach: 1) asthma education is more coherent with a single treatment that addresses both major components of asthma (airway hyperreactivity and inflammation), and which both relieves symptoms and reduces exacerbations; 2) it avoids SABA overuse and the unhealthy dependence on SABA that is associated with asthma mortality and the heavy burden of severe asthma exacerbations seen in LMICs; 3) it ensures greater coverage with ICS, even when patients are poorly adherent [116], because patients receive a controller whenever they use it to relieve symptoms; 4) it provides simpler dose escalation for more severe asthma, and simpler step-down options when symptoms are well controlled; 5) it provides an inbuilt asthma action plan: as asthma worsens, the dose of ICS and formoterol are increased, with both contributing to the reduction in severe exacerbations [117] (studies in both mild [118] and moderate-to-severe asthma [119, 120] show that even a single day of higher ICS–formoterol use reduces the risk of a severe exacerbation in the following 3 weeks); and 6) this single-inhaler ICS–formoterol approach avoids the need for more than one inhaler, thus avoiding complications in LMIC primary healthcare caused by switching of suppliers, dose formulations and inhaler types, which risk overtreatment, under-treatment and confusion about which to use for maintenance and relief. Although these difficulties are not unique to LMICs, they are magnified by the overstressed facilities without adequate support from educators and other support staff.

From the perspective of health services, although the unit cost of medications is currently higher for ICS–LABA *versus* ICS plus SABA, this may be offset by reduced demand for urgent healthcare for asthma exacerbations. It would break the common pattern seen in poorly resourced settings with limited facilities for follow-up of almost total reliance on emergency services for follow-up care. Providing ICS in combination may reduce the need for maintaining the supply chain for some ICS monotherapy formulations and other less effective and more risky asthma medications (such as oral theophylline), which add complexity and cost for facility managers.

However, most evidence for single-inhaler ICS–formoterol therapy comes from HICs, with few LMICs involved in pivotal clinical trials [113, 114, 121, 122]. Pragmatic local trials that include cost–benefit analyses are urgently needed in LMICs to guide policy. If clinically effective, cost-effective and feasible in this context, this simple solution could be implemented on a wide scale. Recent modelling predicted that the use of as-needed ICS–formoterol would be associated with lower cost and reduced disease burden (higher quality-adjusted life years) than maintenance ICS plus as-needed SABA in mild-to-moderate asthma in a middle income country [123].

For patients with mild asthma, where ICS–formoterol is unavailable, GINA recommends that patients should take a dose of low-dose ICS (in a combination or separate inhaler) each time they use their SABA for symptom relief [11]. This strategy is supported by limited evidence from studies in adults [124, 125] and children aged 5–18 years [126, 127], but if separate inhalers are used there is a risk that patients will revert to SABA-only use, particularly where there is no affordable or secure supply of ICS. No studies have assessed ICS–SABA as maintenance and relief, and it may not be suitable for this regimen; in one study, patients randomised to take combination beclometasone–salbutamol regularly twice a day actually had more exacerbations than those receiving twice-daily ICS alone [124].

## What next? Steps to improve asthma care in LMICs

### Achieve a World Health Assembly Resolution on universal access to asthma treatment

Governments must recognise asthma as a priority chronic disease. Health system decision makers must understand that asthma deaths are preventable.

Doctors must insist that it is not acceptable in the present day to manage asthma with SABAs and OCS instead of preventive ICS-containing treatments, emphasising that this common chronic disease can be controlled. The Global Asthma Network has deplored inaccessibility of ICS due to cost, given that “they were developed over half a century ago and their role in cost-effective treatment and prevention of exacerbations and deaths, as well as control of asthma symptoms is well established” [128]. Patients, health workers at all levels of health systems and nongovernment organisations must advocate that all patients should have equitable access to treatments globally acknowledged to be evidence-based and effective.

The research community must develop and evaluate approaches designed to obviate barriers to care in resource-constrained settings. A World Health Assembly Resolution on equitable access to affordable care, including inhaled medicines, for children, adolescents and adults with asthma, wherever they live in the world, would be a valuable step forward, as was achieved recently for diabetes and access to insulin [129].

### Improve healthcare workers’ skills to recognise and manage asthma

Asthma care can be improved through low-cost primary care interventions such as decision support tools embedded in health system-wide programmes [53, 56–65, 107].

Experience implementing PACK shows that asthma outcomes can be improved in LMICs through a programme of case-based ongoing training for primary care health workers, physicians and nurses that is based on adult learning principles and incorporates a well-researched algorithmic disease and symptom-oriented decision support tool [98]. Pharmacists can play an important role in training patients to use inhalers correctly [130] to optimise delivery to the airways.

### Simplify treatment approaches

The dominant current approach to long-term asthma management of maintenance ICS controller plus as-needed SABA reliever has not achieved its potential effectiveness owing to behavioural factors observed in HICs and LMICs. Initiation of treatment with SABA alone encourages patients to rely on SABA; to adhere poorly to ICS owing to cost, perceived lack of effect or fear of side effects; and to seek healthcare only during clinical crises. In LMICs this problem is exacerbated by the suboptimal capacity of clinics to provide the necessary long-term follow-up and self-management education required for effective chronic disease management.

It is therefore necessary to develop and evaluate alternative treatment approaches that are more readily learned and may be preferred by patients [131–133]. Single-inhaler treatment with ICS–formoterol (as-needed ICS–formoterol for mild asthma [113, 114, 121, 122] and MART for moderate-to-severe asthma [109–111, 134–136]) involves the same treatment across all severity levels and requires only dose escalation. Research designed and conducted within LMICs is needed to ascertain its feasibility, clinical effectiveness, cost-effectiveness and acceptability in these populations.

### Conduct research to understand current needs and test new solutions

Comprehensive survey-based research is needed to understand current asthma management across all LMICs. Qualitative research is needed to gain a detailed and deeper understanding of barriers and facilitators to universal access to essential asthma medicines in LMICs. There is also the need for a rigorous programme of implementation research evaluating treatments in the context of the challenges presented in resource-limited settings (*e.g.* case definition, comorbidity and multimorbidity, limited routine record collection, patient mobility, language and health literacy, and overstressed healthcare service personnel). This will involve qualitative studies that examine the appropriateness and cultural acceptability of treatments in each country.

Research questions include the following. 1) What are the benefit and harms of diagnosing and treating asthma syndromically without requiring demonstration of reversible airflow limitation? These are likely to be context-specific, depending on the prevalence of other lung diseases (*e.g.* tuberculosis) and case mix of age (children, adolescents and adults). 2) What are the benefits, harms and cost-effectiveness in LMICs of ICS–formoterol combination inhaler used across the spectrum of asthma severity? 3) What are the most effective in-service methods for training primary care health workers (doctors and other workers) in the diagnosis and management of asthma?

## Conclusion

There are several well-established strategies for the management of asthma in LMICs (and HICs). The inhaled medications needed to provide effective control of asthma have been available for over half a century, are on the WHO Essential Medicines List and can be produced at a very low cost. The implementation of any of these strategies incorporating access to affordable quality-assured inhaled asthma medications (including ICS) should be prioritised.

## Shareable PDF

10.1183/13993003.03179-2021.Shareable1 This one-page PDF can be shared freely online.
Shareable PDF ERJ-03179-2021.Shareable
