# Supplementary material for: Asthma management in low and middle income countries: case for change
Source: Eur Respir J. 2022 Sep 15;60(3):2103179. doi: 10.1183/13993003.03179-2021 (PMC9474897; doi:10.1183/13993003.03179-2021)
Supplement: Supplementary file 1 [file ERJ-03179-2021.Shareable.pdf]

SHAREABLE PDF

# Asthma management in low and middle income countries: case for change

**Kevin Mortimer [1,2], Helen K. Reddel [3], Paulo M. Pitrez [4] and Eric D. Bateman [5]**

[1]Dept of Respiratory Medicine, Liverpool University Hospitals NHS Foundation Trust, Liverpool, UK. [2]Dept of Medicine, University of Cambridge, Cambridge, UK. [3]The Woolcock Institute of Medical Research and The University of Sydney, Sydney, Australia. [4]Pediatric Respiratory Division, Hospital Moinhos de Vento, Porto Alegre, Brazil. [5]Division of Pulmonology, Dept of Medicine, University of Cape Town, Cape Town, South Africa.

Corresponding author: Kevin Mortimer (kjm20@cam.ac.uk)

Check for updates

Shareable abstract (@ERSpublications)
**Many children, adolescents and adults with asthma in LMICs lack access to effective and affordable asthma care that would improve and save lives. A World Health Assembly Resolution on access to effective and affordable asthma care for all is needed.** https://bit.ly/3gLquDa

**Cite this article as:** Mortimer K, Reddel HK, Pitrez PM, *et al*. Asthma management in low and middle income countries: case for change. *Eur Respir J* 2022; 60: 2103179 [DOI: 10.1183/13993003.03179-2021].

This single-page version can be shared freely online.

Copyright ©The authors 2022.

This version is distributed under the terms of the Creative Commons Attribution Licence 4.0.

Received: 16 Dec 2021
Accepted: 7 Feb 2022

## Abstract

Asthma is the most common noncommunicable disease in children, and among the most common in adults. The great majority of people with asthma live in low and middle income countries (LMICs), which have disproportionately high asthma-related morbidity and mortality. Essential inhaled medications, particularly those containing inhaled corticosteroids (ICS), are often unavailable or unaffordable, and this explains much of the global burden of preventable asthma morbidity and mortality. Guidelines developed for LMICs are generally based on the outdated assumption that patients with asthma symptoms <1–3 times per week do not need (or benefit from) ICS. Even when ICS are prescribed, many patients manage their asthma with oral or inhaled short-acting $\beta_2$-agonists (SABA) alone, owing to issues of availability and affordability. A single ICS–formoterol inhaler-based approach to asthma management for all severities of asthma, from mild to severe, starting at diagnosis, might overcome SABA overuse/over-reliance and reduce the burden of symptoms and severe exacerbations. However, ICS–formoterol inhalers are currently very poorly available or unaffordable in LMICs. There is a pressing need for pragmatic clinical trial evidence of the feasibility and cost-effectiveness of this and other strategies to improve asthma care in these countries. The global health inequality in asthma care that deprives so many children, adolescents and adults of healthy lives and puts them at increased risk of death, despite the availability of highly effective therapeutic approaches, is unacceptable. A World Health Assembly Resolution on universal access to affordable and effective asthma care is needed to focus attention and investment on addressing this need.

CC BY
